# How to stop being surprised by unprecedented weather

**DOI:** 10.1038/s41467-025-57450-0

**Published:** 2025-03-10

**Authors:** Timo Kelder, Dorothy Heinrich, Lisette Klok, Vikki Thompson, Henrique M. D. Goulart, Ed Hawkins, Louise J. Slater, Laura Suarez-Gutierrez, Robert L. Wilby, Erin Coughlan de Perez, Elisabeth M. Stephens, Stephen Burt, Bart van den Hurk, Hylke de Vries, Karin van der Wiel, E. Lisa F. Schipper, Antonio Carmona Baéz, Ellen van Bueren, Erich M. Fischer

**Affiliations:** 1 Climate Adaptation Services Foundation (CAS), Bussum, The Netherlands; 2 Institute for Environmental Studies, Vrije Universiteit Amsterdam, Amsterdam, The Netherlands; 3 Red Cross Red Crescent Climate Centre, The Hague, The Netherlands; 4 Department of Meteorology, University of Reading, Reading, UK; 5 Royal Netherlands Meteorological Institute (KNMI), De Bilt, The Netherlands; 6 Deltares, Delft, The Netherlands; 7 School of Geography and the Environment, University of Oxford, Oxford, UK; 8 Institute for Atmospheric and Climate Science, ETH Zurich, Zurich, Switzerland; 9 Institut Pierre-Simon Laplace, CNRS, Paris, France; 10 Geography and Environment, Loughborough University, Loughborough, UK; 11 Feinstein International Center, Friedman School of Nutrition Science and Policy, Tufts University, Boston, MA USA; 12 Department of Geography, University of Bonn, Bonn, Germany; 13 University of St. Martin (USM), Philipsburg, Sint Maarten; 14 Faculty of Architecture and the Built Environment, Delft University of Technology, Delft, The Netherlands

**Keywords:** Climate-change impacts, Climate-change adaptation

## Abstract

We see unprecedented weather causing widespread impacts across the world. In this perspective, we provide an overview of methods that help anticipate unprecedented weather hazards that can contribute to stop being surprised. We then discuss disaster management and climate adaptation practices, their gaps, and how the methods to anticipate unprecedented weather may help build resilience. We stimulate thinking about transformative adaptation as a foundation for long-term resilience to unprecedented weather, supported by incremental adaptation through upgrading existing infrastructure, and reactive adaptation through short-term early action and disaster response. Because in the end, we should take responsibility to build resilience rather than being surprised by unprecedented weather.

## Abstract (web summary)

Unprecedented weather events are increasingly impacting societies worldwide. This Perspective explores methods to anticipate such hazards, and it highlights the role of transformative, incremental, and reactive adaptation strategies to achieve enhanced resilience.

## Introduction

There are different gradients of extreme weather - from the average hot day to the record-breaking heatwave, from the annual springtime flood to the unprecedented dam breach, from an average tropical cyclone to one occurring after the season is meant to be over. For example, in September 2017, Hurricanes Irma and Maria damaged approximately 95% of the buildings and forced thousands of residents to move into public shelters on the island of Sint Maarten/Saint Martin^1^. In neighbouring Puerto Rico, the same hurricanes accounted for more than 4600 deaths, mostly in connection with poor public health infrastructure and essential public services^2^. In 2020, the Horn of Africa recorded its fifth consecutive failed rainy season, with poor pasture conditions, livestock losses, decreased surface water availability and human conflicts, leaving 4.35 million people in need of humanitarian assistance^3^. In October 2021, severe floods in southwestern Nepal were unprecedented because they occurred outside of the usual monsoon season, catching warning systems off guard and disrupting agricultural activities, causing over 120 deaths and the displacement of over 4790 families^4^. In July 2021, the Pacific Northwest of North America saw temperatures soar above 45 °C, shattering records for the region. Lytton, BC, Canada, experienced an especially severe spike, with temperatures reaching 49.6 °C, 5.2 °C higher than the previous record set in 1941 from observations dating back to 1917. This event strained the healthcare systems and resulted in over 850 deaths^5^. All these events were unprecedented in different ways but had devastating impacts^6^.

It is common to encounter media narratives emphasising the surprise caused by unprecedented weather. Corresponding gaps in disaster preparedness systems and adaptation actions leave communities underprepared and unequipped to handle “surprising” weather events^6,7^. However, advances in climate science of various kinds are rapidly increasing our understanding of current and future risks of unprecedented weather, and can be used to reduce their impacts by informing disaster management and climate adaptation practices.

The overarching aim of this perspective piece is to argue that we can avoid being surprised by unprecedented weather and ensure that it does not cause unprecedented impacts when it occurs. In this piece, we provide the first comprehensive overview of approaches for analysing unprecedented weather. We then discuss how these approaches can inform disaster management and climate adaptation practices to build resilience to unprecedented weather and ensure that it does not cause unprecedented impacts.

## Approaches for analysing unprecedented weather

**Fig. 1:**
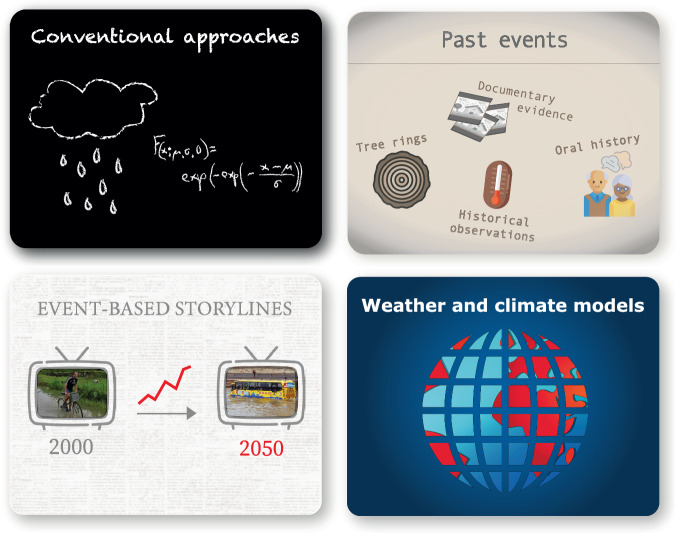
An overview of the categories of approaches that exist to identify unprecedented weather. The thermometer icon was made by justicon; tree rings and oral history icons made by Freepik and flood icon made by Konkapp and obtained from www.flaticon.com.

Approaches to identifying unprecedented weather can be divided into four categories based on the complementary lines of evidence they may provide: conventional methods, past events, event-based storylines, and weather and climate-model data exploration (Fig. 1, Table 1). We refer to Baldisarri Pacchetti et al.^8^ for a discussion on the typology of evidence for regional climate information.

**Table 1:** An overview of the four lines of evidence to identify unprecedented weather hazards discussed here, and their key benefits and limitations

Lines of evidence	Benefit	Limitation
**Conventional statistical methods using observations**	Represent events that actually occurred	Are inherently limited in identifying unseen events
**Past events from historical observations, documentary evidence, oral history and proxy data**	Help understand events that may have happened before the modern observational records	Are rare, may be challenging to compare to present conditions, and (for the proxy data) have limited resolution
**Event-based storylines**	Provide a physically plausible unfolding of a single event	Only applies to the single event
**Weather and climate-model data exploration**	Produce information outside what is possible from observations	Rely on the realism of these models

These categories follow a chronological order, starting with conventional methods and studying past events, which have been widely used, and progressing to the more recent approaches from storylines and weather and climate models. Conventional approaches primarily utilise measurements of weather variables. Past events can be based on historical observations, documentary evidence, oral history and proxy data. Event-based storylines can be constructed from a variety of data sources, combining climate modelling with expert judgement, historical observations, proxies, and physical understanding. Weather and climate-model-based approaches depend on the outputs of physics-based models.

These four lines of evidence have specific benefits and limitations, outlined in Table 1.

### Conventional statistical approaches using observations

There is a long history of science informing society about extreme weather events that have not been experienced before. Statistical modelling of extreme values based on weather records became possible around the 1950s^9^. Its use was quickly taken up by engineers for the estimation of flood design values^10,11^. By the end of the 20th century, weather generators were developed that simulate long synthetic series^12^. They have been widely used in engineering design^13,14^. This section describes these conventional approaches, including their more recent developments.

*Extreme value statistics* is the conventional method to estimate the probability distribution of extreme events^9,15^. This method works by choosing an Extreme Value distribution and fitting it to available observational data. Extreme events beyond the observational record can then be estimated by extrapolation, for a given return period, based on the fitted distribution. It is possible to further account for the non-stationarity of the climate system, e.g., in a warming climate, and to estimate how the statistics of extreme events have changed with time or global temperature^16^. The benefit of extreme value statistics is that this approach has been widely tested, used, and adopted for estimating design values. The strength of the approach using observations is that it is grounded in reality. The major limitation of using observed records is their relative brevity and sparsity, which can lead to large uncertainties when estimating an extreme event, especially if the desired return period of the estimated event exceeds the length of the observed record. Short observed records of several decades may lead to a systematic underestimation of extreme events^17^, which may make unprecedented weather appear impossible using extreme value statistics^18^. Other limitations include the susceptibility to measurement errors^19^, data inhomogeneities, spatial aggregation (local observation points may not be representative of large events), the dependence on the choice of distribution, sensitivity to the tails of the distribution, the assumption of independent events, and that these methods are limited by what was plausible in a past climate given that they are based on what has happened in the past. In the future, climate events may become more extreme because the mechanisms behind them may change, leading to a different distribution altogether.

*Spatially pooling observation stations* is one option to increase the observational sample size^20,21^, noting this may introduce other complexities such as dependence between the observation stations. More sophisticated approaches include spatial statistical models based on extreme value theory^22,23^. A ‘*near-miss’ event* is sometimes used as a simple observation-based alternative from a nearby station to assess the potential impacts of a historical event that might plausibly occur in a target location. For example, De Bruijn et al.^24^ studied the potential impacts of the heavy central European rainfall event in 2021 if it had been located over different regions in The Netherlands with different hydrological response characteristics to the affected region. However, such approaches need to make assumptions about whether the same events are plausible at other spatial locations, which is particularly challenging in complex topography, along complex coastlines or heterogeneous land use conditions.

*Weather generators* are another widely adopted method to increase the sample size by statistically emulating weather events^12,25–29^. Weather generators also fit a chosen distribution to the historical data. The main difference with extreme values statistics is that while extreme value theory targets just the tails (extremes) of the distribution, weather generators are trained on the entire distribution of events. The strengths of weather generators for estimating extreme weather events include the ability to produce much longer sequences of data than observations; to reproduce temporal/spatial correlation structures^28^; to incorporate different covariates (such as large-scale atmospheric conditions^27^) to condition the distribution on a subset of events^30^; and to incorporate multi-variate dependency and assess compound extremes^26^. The main challenge for weather generators is producing plausible statistical properties of observed extremes and capturing their interactions given the limited length of observed records. Statistical weather generator accounting based on atmospheric circulation analogues has recently been extensively used to estimate the potential intensity of very rare heat and cold waves^31^.

*AI and machine learning* models can also support efforts in anticipating and modelling high-impact and unprecedented weather. One of the main current uses of AI models is in weather forecasting, where they have the potential to achieve better performance, longer lead skills and run at much faster speeds than traditional physics-based models. For example, AI-based forecasting models have been shown to outperform traditional numerical models in some metrics, potentially improving early warning systems and disaster preparedness^32,33^. However, there are other examples where physics-based models still perform better for an extreme event^34^ and it remains unclear how skilful these methods are in extrapolating to unprecedented events outside their training datasets. One challenge with these approaches is identifying the drivers of unprecedented weather events, noting that advances in explainable AI may offer promising avenues to address this challenge^35^.

### Past events: historical observations, reanalyses, documentary evidence, oral history and proxy data

The ‘modern’ era of observations (after about 1950) is the best sampled period available and includes the recent decades of the most rapid global warming. However, this ~70-year period is short when analysing unprecedented weather. This section describes approaches to estimating and understanding historical events before this modern era of observations. Longer records can help understand weather events that otherwise would be deemed unprecedented^36–38^. It can be challenging, however, to directly compare past weather to present conditions, for example, due to changes in land use and infrastructure affecting the impacts of the events, or because of the larger uncertainty which could lead to an over-representation of apparent weather events in the distant past that exceed modern records.

**Fig. 2:**
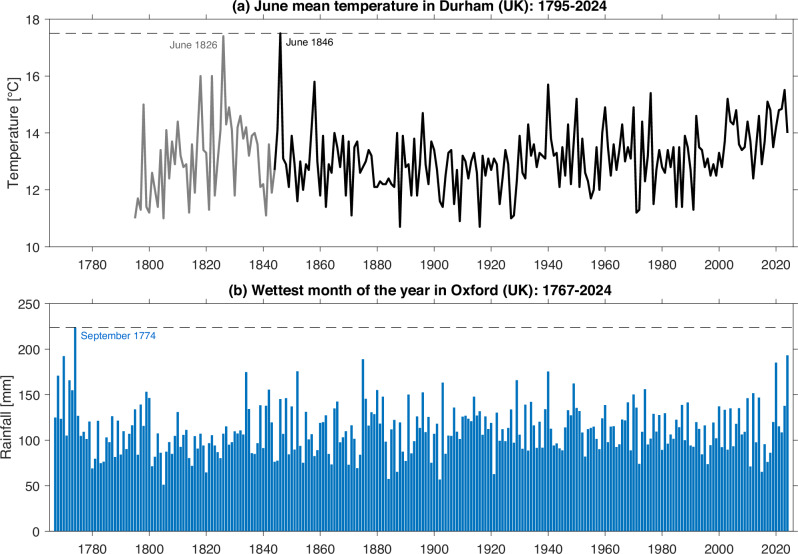
Examples of historical weather events which are unsurpassed. In Durham (UK), the two warmest Junes on record occurred in 1826 and 1846, and are >1.5 °C warmer than any other June in the post-1850 period (**a**). The grey lines indicate times when only monthly data are available, whereas daily data are available for the period shown by the black line. For Oxford (UK), the wettest month on record remains September 1774, around 15% wetter than any other month (**b**); recently, September 2024 became the second-wettest on the 250+ year record.

Although *direct instrumental observations* of past weather events that would be unprecedented in the modern era are rare, they are immensely valuable as they represent events that actually occurred. Two striking examples are shown in Fig. 2.

Figure 2a shows the mean temperature for June in Durham, in north-east England, back to 1795^39,40^. Although June 2023 became Durham’s hottest June since 1940, it is clear that there have been significantly hotter Junes in the past, notably those in 1826 and 1846. Even making careful allowance for uncertainty in temperature records before the modern era^41^, the two historical events indicate that monthly mean temperatures at least 1 °C hotter than temperatures recorded in June 2023 have occurred outside the period of modern climate records. Had the same temperatures of June 1846 occurred in June 2023 without the availability of the pre-1850 records, it may have been labelled a record-breaking (or even record-shattering) event, but the longer records enable us to show the full context.

Figure 2b shows the wettest month in each calendar year at Oxford’s Radcliffe Observatory site since 1767^42^. Recently, September 2024 became Oxford’s wettest month in over 200 years with 193.3 mm – such an extraordinarily anomalous month would, for almost anywhere else in the world, appear to be a record-breaking event, but it is still not quite the greatest monthly fall at this very long-period site. In this continuing 250+ year record^42^, September 1774 remains by far the wettest month on record, with around 15% more rainfall than any other month. Although the 1774 rainfall amount appears to be implausible at first sight (particularly since September is rarely the wettest month in any given year), it is well-supported by other independent rainfall measurements and contemporary diaries, together with well-documented severe flooding throughout the river Thames and other catchments. Although the gap between Oxford’s ‘wettest-ever’ and ‘second-wettest-ever’ months has reduced very slightly, when it comes to planning water resource management it is still helpful to be able to indicate - based on clear observational evidence - that a month could still be at least 15% wetter than any experienced in recent times.

Large numbers of existing historical records are yet to be digitised, including meteorological and phenological observations, data from tide gauges, and river flow measurements. The ‘rescue’ of such data from paper or magnetic tape archives can provide valuable information about past events^43^. For example, in the UK, the driest year on record was recently revised downwards because a lower total rainfall year (1855) was identified through recovering additional and longer observational records by volunteer citizen scientists^44^.

Observations are also used to help build *reanalyses* of past weather conditions. These exist for the modern period^45,46^, on centennial timescales^47^ and even multi-centennial timescales^48^. These datasets blend observations with weather forecast models to produce spatially and temporally coherent outputs, i.e., ‘maps without gaps’^45^ which can be used to consider extreme events and how they are changing. For example, rainfall intensity-duration-frequency (IDF) curves have been estimated globally^49^, and past extreme events have been credibly reconstructed, e.g., a blizzard in 1888^50^ and an intense windstorm in 1903^51^. However, they are limited by the availability of observations to constrain the atmospheric circulation, which become more sparse further back in time.

*Documentary evidence* for past events, in the form of written observational records, photographs, or drawings, can also provide context and show that recent events are sometimes not as unprecedented as they may first appear. For example, the river Ahr (a tributary of the Rhine in Germany), which experienced severe flooding in 2021, also saw extreme floods in 1910 and 1804^52^.

*Oral history* of past events is another source rooted in local and Indigenous knowledge^53^, offering another perspective than can be obtained from data^54–56^, providing a memory which may not exist in observational records. Oral traditions, often shared across generations, provide unique insights into past experiences of extreme weather, highlighting ethical perspectives and cultural values^57^ that are deeply intertwined with environmental knowledge^58^. Beyond identifying unprecedented weather through local forecast knowledge^59^, these narratives convey how people have learned to cope with extreme weather^60–62^. In the context of building resilience to unprecedented weather, oral history thus does not only offer past experiences but also embodies place-based wisdom for culturally appropriate responses^63,64^.

*Proxy-based methods* can sample rare extreme weather events that may have occurred prior to (and hence are unprecedented in) the modern instrumental data record by reconstructing information from ice cores, coral reefs, speleothems, pollen, ocean sediments, tree rings, and diatoms^65–69^. Limitations of proxy-based methods are the dating uncertainty; the coarse spatial-temporal resolution relative to direct observations (e.g., decades, centuries or longer); methods are calibrated for the bulk of the distribution and not intended to capture the tails; sensitivity to other signals; degradation of the proxy record’s information content; and the inhomogeneous coverage of reconstructions of extremes^70^. These methods are therefore well-suited for large-scale and long-duration event types, such as droughts^66,71^, seasonal temperatures over large domains^68,72^ and also certain large floods^73,74^. Novel approaches also include daily to hourly resolution biogeochemical proxy records^75^, but limitations are substantial and cannot be easily overcome on these timescales. There are also multi-millennial reanalyses which combine climate-model simulations and proxy information to produce global estimates of climatic changes^76^.

### Event-based storylines as an alternative to probabilistic approaches

Storylines consist of physically plausible and self-consistent unfolding of climatic events, typically based on historical (high-impact) events. Storylines focus on plausibility and understanding of the driving factors instead of the occurring probability of such events^77–79^. This enables the exploration of alternative realisations of the historical event (also known as counterfactuals) through documented changes in the driving conditions, which can include climate change and climate variability information. Examples of the use of storylines to investigate unprecedented weather and its impacts across different fields include floods^80^, tropical cyclones^81,82^, storms^83^, crop failures^84^, droughts^85–87^, among others.

*Pseudo Global Warming (PGW) and nudging approaches* simulate historical or recent events under alternative scenarios, such as past, present or future climates. Although extreme events are unique and unlikely to reoccur in exactly the same way, this method provides a powerful communication tool, allowing plausible future events to be linked to lived experiences. One common approach uses high-resolution regional climate-model simulations: one of the events in the current climate using reanalysis as boundary and initial conditions, and future and/or past simulations of the event where a mean change signal is added to the boundary and initial conditions^88–90^. Some studies adapt the method to allow for future changes in large dynamics beyond a mean change^91,92^. Another technique takes a weather forecast model and reruns with the ocean temperatures and CO_2_ concentrations altered to create past and future counterfactuals^93,94^. Nudging of the atmospheric circulation to impose the dynamical conditions of a specific event can also be applied, for example through large spectral nudging allowing only large-scale features to be constrained^95–97^. A related approach uses two reanalyses, assimilating the same surface pressure observations and only differing by the SST boundary conditions, to translate specific events into different climates^98^. In these approaches, the structure of the atmosphere is altered - perhaps away from the unique event being assessed. These differences may lead to changes in, e.g., rainfall, thus care must be taken in interpreting results. The development of similar methods by different research groups presents an opportunity to compare results and better understand the limitations and advantages of each. To ensure storylines are relevant for society, the selection of events, the scope of the storylines, and the changes in boundary conditions can be informed by stakeholders' input and/or through investigation of historical high-impact events, including their drivers and sectoral impacts^99^.

*Ensemble boosting* is another method that may provide input for physical climate storylines^100,101^. Ensemble boosting uses targeted re-initialisation of very extreme events in large ensembles of fully coupled climate models. Thereby, large ensembles of extreme events in the climate-model can be produced in order to efficiently sample the most extreme events. Ensemble boosting has been applied to quantify and understand potential future multi-year droughts^102^, heavy-precipitation events^103^ or very cold winters^104^. Additional challenges arise for small-scale events that have shorter predictability timescales and require kilometre-scale models, and the suitability of the ensemble boosting for such types of events requires further testing.

Storylines have the benefit of providing locally relevant information that can directly be integrated into decision-making^77,79,99^. In addition, storylines improve people’s risk awareness, as people react more directly to memorable events than to a range of future possibilities represented by probabilities^78,105,106^. Limitations include the potential that storylines may not be accepted when produced without local involvement, may miss other plausible unprecedented weather events outside the predefined conditions of the storyline being produced, and that certain decision-making processes, like disaster risk financing mechanisms, require probabilistic information^107^.

### Weather and climate-model data exploration

The work from Lorenz^108^ was pivotal in the suite of approaches for simulating unprecedented weather based on weather and climate-model simulations. Lorenz explained the limitations of weather predictions induced by the chaotic nature of the atmosphere. By making very small changes in the initial conditions of weather simulations, akin to a butterfly effect, a range of possible weather outcomes is obtained, representing different plausible evolutions of future weather. This presents an opportunity to explore a realistic plausibility range for future weather events^109,110^. With recent computational advancements and open-source programming^111^, along with the increased attention to high-impact events that occur around the world, this field of science has grown rapidly.

This category of approaches for estimating unprecedented weather gathers the outputs of multiple weather or climate-model simulations to produce a large sample size, relative to historical observations. The difference with previously mentioned weather generators is that these model simulations are based on fundamental principles of physics to simulate numerically the interactions between the atmosphere, ocean, land, and ice, and thus preserve physical consistency across variables and across space and time while weather generators rely more on statistical methods to generate possible weather scenarios. The difference with event-based storylines is that here, we discuss analysing existing weather and climate-model data through search and data-mining techniques, as compared to targeted model-based experiments of individual events.

The benefit of this line of evidence is that it may provide information about physically plausible events that lie outside the range of observed events, and the types of processes that could lead to unprecedented weather. The approach hinges on the limitations of weather and climate models to simulate unprecedented weather realistically. Moreover, the application of bias adjustment can help to compensate for errors between modelled and observed baseline climatology, however, it can also lead to the generation of spurious extremes – such as when uplifting precipitation amounts and thereby runoff events to unrealistic levels^112^ and hamper the physical consistency across variables^113^. This section describes the types of models that can be used, their benefits and limitations, and other similar types of model experiments that have been used for studying unprecedented weather.

*Operational weather forecasting* centres store large archives of past weather forecasts and seasonal or decadal climate predictions which can be exploited to assess events that have been simulated but not yet occurred. Van den Brink et al.^109^ was the first to use probabilistic weather predictions to explore plausible unprecedented weather, after which the approach was popularised as the ‘*UNSEEN*’ method: the UNprecedented Simulated Extremes using ENsembles^110,111^. The strengths of this approach can include increased resolution relative to climate models, the inclusion of additional processes, the ongoing forecast evaluation at weather centres, and the positive impact of the initialisation to reproduce observed weather and climate phenomena^114^. Key limitations include: events in future climates cannot be studied, that generally numerical weather and climate prediction models have systematic biases depending on the forecast lead time, and that these forecasts represent a limited sample of plausible ocean conditions.

*Climate-model projection* systems are better suited for understanding unprecedented weather under different climate scenarios and due to the fully coupled atmosphere-ocean interactions also sample ocean and sea ice conditions that have not been recently observed^115^. The most commonly used models for projecting climate change are available through the Coupled Model Intercomparison Project^116^. There always has been a trade-off between the resolution of the model and the number of ensemble members that can be used to increase the sample size for detecting extremes, because of the large computational power that weather and climate models require. There is also a trade-off between using a large collection of models and the risk of including biased climate projections. A multi-model ensemble introduces the difficulty of disentangling model uncertainties from the irreducible uncertainty of outcomes that can happen in the real world. To this end, a *Rare Event Algorithm* has been developed to improve the statistics of extreme events for simulations that are not long enough to sample rare events^117,118^.

*High-resolution climate-model experiments* have been simulated for global models through the High-Resolution Model Intercomparison Project^119,120^ and for regional models through the Coordinated Regional Climate Downscaling Experiment CORDEX^121,122^. *CORDEX* offers more reliable information compared to other climate-model experiments about some local-scale sub-daily extremes, such as convective precipitation and wind gusts, allowing the study of future hydro-meteorological extremes^121,123–125^. A limitation of CORDEX is the small sample size, restricting reliable extreme value estimation. At even higher resolution convection-permitting models improve realism in the simulation of local-scale sub-daily extremes^126^ but are hampered by very limited availability of multi-member and multi-model experiments^127,128^.

*Large numbers of ensemble members* have been produced in Single Model Initial-condition Large Ensembles experiments (*SMILE*s^129,130^). These have the key benefit of extensively sampling climate variability in historical simulations and future climate projections. This developing field of science facilitates the analysis of unprecedented weather with large sample sizes and more robust statistics^131^, but the resolution of the climate models used might be too coarse to represent all relevant processes and climate extremes. SMILEs can be dynamically downscaled with regional climate models to produce high-resolution large ensembles^123,132–134^. Despite the coarse resolution, they may be useful to robustly estimate changes and study future events, especially those less affected by lack of high resolution like temperature and drought extremes^85,86,135,136^.

### Using multiple lines of evidence

All of these methods have their benefits and limitations: conventional statistical methods using observations represent events that actually occurred but are inherently limited in identifying unseen events; past events from historical observations, documentary evidence, oral history and proxy data are immensely valuable in helping understand events that may have happened before the modern observational records but are rare, may be challenging to compare to present conditions, and (for the proxy data) have limited resolution; event-based storylines provide a physically plausible unfolding of a single event, but only for a single event; and weather and climate-model data explorations produce information outside what is possible from observations but rely on the realism of these models. As such, these methods may present complementary lines of evidence. Box 1 illustrates the use of four complementary approaches to identify plausible unprecedented heat near the city of Eindhoven, the Netherlands.

Box 1 Unprecedented heat in The Netherlands: exploring multiple lines of evidence Eindhoven is a large city in the south of the Netherlands, situated in the region where on 25 July 2019 the 40 °C-threshold was breached for the first time - the highest recorded temperature in the country since records began. Eindhoven is selected as a case study to illustrate the use of four complementary approaches to identify plausible unprecedented heat in this region (methods described in the Supplementary Materials). The conventional approach shows that there is a large trend in the highest temperatures between the pre-industrial and present climate (Box 1a). Proxy-based methods suggest that the summer of 1540 was most anomalous (Box 1b), supported by previous studies^68,137–139^. The storyline approach shows that the July 2019 heatwave would reach up to 45 °C in a 2 °C warmer world (Box 1c). Climate model projections show temperatures of 48 °C near the end of the century under a high-end scenario, noting these are likely underestimates (Box 1d). As such, the methods provide complementary information, together showing strong evidence for the potential of unprecedented heat near Eindhoven beyond that in the conventional observational record.

**Figure:**
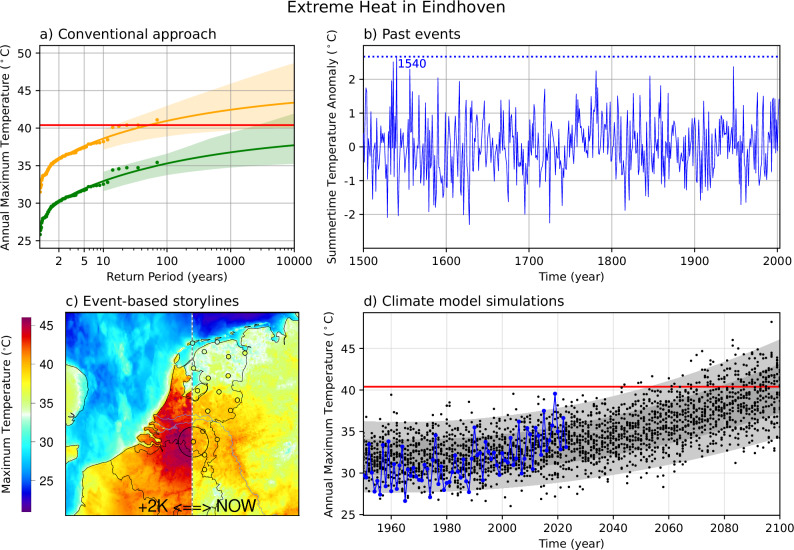
**a** Annual daily maximum air temperature at Eindhoven, 1951–2023 (95% CI) with the effects of global mean surface temperature (smoothed) linearly subtracted from the position parameter, for the preindustrial (in green) and current climate (in orange). The observed record temperature of 40.4 °C in 2019, is shown by the red horizontal line (Data is from the KNMI observational network, station number 370). **b** Reconstructed temperature data for summertime (June–July–August) mean temperature anomalies over the Netherlands (3.75–6.75 °E, 51.25–53.75 °N) from 1500 to 2003 (Data taken from KNMI Climate Explorer, as in Luterbacher et al.,^139^). **c** Maximum temperature during the three hottest days of the July 2019 heatwave as simulated by the convection permitting climate model HCLIM43 for the current climate (right half) and under +2 K warmer scenario (left half). The small circles in the right half show observations from Dutch Meteorological stations. The large circle is centred around the city of Eindhoven. **d** Annual daily maximum air temperature at the nearest grid point to Eindhoven in ERA5 (blue dots) and in a bias-adjusted 16-member regional climate model ensemble (black dots). The regional climate model RACMO was nested in the global climate model EC-Earth3; historic forcing until 2014, SSP5-8.5 forcing from 2015. Grey shading shows an estimated normal distribution (mean plus 1 and 2 standard deviations) based on the model ensemble.

## How to stop being surprised

We can be surprised by unprecedented weather or unprecedented impacts. To stop being surprised by the hazard, the “Approaches for analysing unprecedented weather” section has discussed scientific advances which could be applied to help anticipate unprecedented weather. To stop being surprised by the impacts, in this section we describe disaster management and climate adaptation approaches to build resilience. These approaches can be seen as three types of adaptation: reactive, incremental, and transformative adaptation^140^. These types relate to various components of disaster management: reactive adaptation relates to short-term disaster preparedness and response actions; incremental adaptation refers to long-term disaster prevention; while transformative adaptation relates to transformational risk management and goes beyond reactive and incremental adaptation by aiming to reshape and fundamentally alter the systems, structures, and practices to achieve more just and equitable outcomes^141^.

**Fig. 3:**
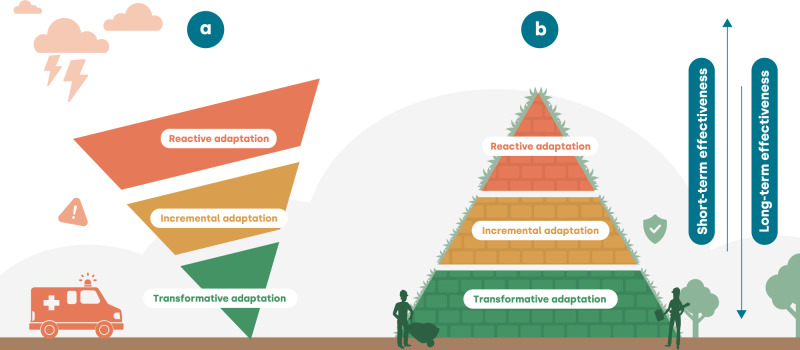
The conceptual adaptation pyramid illustrates how three layers of adaptation contribute to resilience to unprecedented weather. **a** An unstable pyramid: with little transformative actions, we increase our reliance on incremental adaptation and place a lot of emphasis on handling unprecedented weather through early warning, early action and disaster response. **b** A stable pyramid: with more emphasis on transformative actions, we may be able to prevent limits to incremental adaptation, and have to place less emphasis on reactive actions.

Identifying the right mix of measures is important for preparing for unprecedented weather. We use the conceptual ‘adaptation pyramid’ to illustrate how the three different types of adaptation, depicted in three layers, may influence our ability to handle unprecedented weather (Fig. 3). The adaptation pyramid symbolises a climate-resilient system and was developed as a visual instrument and method for adaptation policymakers. This pyramid approach was recently adopted by the national Delta Program in the Netherlands for setting local adaptation goals and targets^142^.

Here, we use the pyramid as a concept to build resilience to unprecedented weather. The conceptual adaptation pyramid may help stimulate thinking about transformative actions being the foundation to ensure resilience to unprecedented weather in the long term, with residual risks captured through incremental adaptation and reactive adaptation. Yet, as transformative actions take time, reactive adaptation and short-term disaster preparedness and response actions may be equally important for unprecedented weather that may happen in the near future. We should therefore identify the right mix of actions we can take over time. For each of the layers discussed below, we describe the current practices, gaps, and advances related to the approaches described in the “Approaches for analysing unprecedented weather” section (Table 2).

**Table 2:** A summary of the current practices for each of the layers of adaptation, gaps, and advances related to the approaches described in the “Approaches for analysing unprecedented weather” section, including the discussed case studies

Adaptation layer	Current practice	Gap	Advances	Cases
**Reactive**	Weather prediction	Limited trust in unprecedented weather forecasts	Putting forecasts of unprecedented weather in perspective	October 2021 Nepal Floods
	Scenario planning	Often designed based on experience with past events	Developing scenarios of unprecedented weather	-
**Incremental**	Stress testing infrastructure	May under-appreciate the risk of unprecedented weather	Stress-test systems to unprecedented events	UK water security
				Disruption of food security in the Horn of Africa
**Transformative**	Climate-Resilient Development	Not directly supported by advances in physical climate science	Switches focus from the impacts and hazards towards the underlying development context and drivers of vulnerability	2017 Hurricanes Irma and Maria

### Reactive adaptation and short-term disaster preparedness

One of the most widely used climate services is weather prediction, which operationally informs early warnings and early action to extreme weather events^143^. By doing so, these predictions help to reduce the impacts from weather extremes and prevent them from escalating into disasters.

However, a gap with the current warning systems is that when there is a warning of something out-of-the-ordinary in terms of severity, duration, and/or timing, some may perceive it as impossible and therefore not take proportionate action on the warning received^144^. Research has shown that trust in early warning is determined by experience and perception of risk at that particular moment and may be heightened in high-risk periods or seasons to lead to action^145–148^. For example, during the October 2021 floods in Nepal, anecdotal evidence suggests that the warnings that were issued to the public were also not as effective as they could have been because they were initially treated with disbelief that a flood could happen that late in the year^4,149^.

Applying the multiple lines of evidence described above to identify unprecedented weather may help to put those forecasts in perspective. This may inform new gradients of severity for early warnings of forecasted unprecedented weather, or the use of special wording (e.g., through analogues of past events). Whilst research on early warning messaging is significant, more efforts may be placed on the alteration of the content of standard early warning messages for unprecedented weather. Efforts in the research and practice communities are being made to centralise the value of local and Indigenous knowledge about weather and climate and find ways to integrate these knowledge systems and “scientific” forecasts^150–152^. Particularly for extreme weather, it can be critical to increase the reliability, usefulness, and use of weather predictions at different scales.

Disaster managers often make use of tools such as scenario planning and simulations to prepare disaster response to extreme events^153–155^. Desktop and active simulations are favoured tools for this whereby practitioners are given a disaster scenario to respond to, sometimes in real-time; discussions and learnings are captured along the way, and these help inform necessary changes or tweaks to contingency plans and future response operations^156^. A gap with these scenarios is that they are often designed based on experience with past events, and may not reflect risks from unprecedented weather.

Various approaches to identify unprecedented weather can be used in developing the scenarios. For instance, emergency managers can use such scenarios to expand their understanding of plausible unprecedented weather and use this in their planning as well. This information could then be used in contingency plans to respond to these events if/when they occur.

### Incremental adaptation

Incremental adaptation maintains the essence and integrity of a system or process at a given scale^157^. Incremental adaptation, for example, involves making gradual updates and improvements to existing infrastructure to enhance resilience against climate impacts. Stress testing frequently serves as a method to evaluate how infrastructure can be improved to reduce climate risks to an acceptable level^158^. The use of stress testing is most beneficial in highly precautionary circumstances or when critical infrastructure ‒ such as health care, energy and water supplies ‒ could be disrupted. For example, designs for long-lived coastal assets such as ports, flood defences, transport routes, or (nuclear) energy may be stress-tested against a high-end storm, surge, and wave combinations^83,159,160^.

Climate-model scenarios and conventional statistics are commonly used to calculate these climate risks. However, a gap in using these conventional approaches is that they may under-appreciate the risk of unprecedented weather. The advances in the approaches described in the “Approaches for analysing unprecedented weather” section may help to stress-test systems to unprecedented weather. For example, in the UK, the resilience of water supply-demand systems has to be assessed for a 1 in 500-year drought. Previously, this has been done using weather generator techniques that can be perturbed to represent climate variability and change. However, large climate-model ensembles can also give likelihoods of problematic conditions for water companies – such as successive dry winters in lowland England^85,161^. Recent observational data rescue activities have also newly identified a period during the 1850s when UK rainfall was the lowest on record^44^. These unprecedented weather events can also be used to stress-test the efficacy of various strategic water resource options, including water demand management^162^.

Storylines can be developed to explore alternative scenarios of unprecedented weather events, such as the effects of concurrent events on the disruption of food security in the Horn of Africa^99,163^. Using as reference the 2019/21 locust infestation that led to food shortages in that region, counterfactual scenarios are built by combining this event with the multi-breadbasket failures of 2007/08 and the subsequent crop export restrictions that occurred during that same crisis. Results show a considerable increase in impacts, with many countries in the region having food imports reduced by one-third and unable to compensate for the shortages with their own stockpiles. This experiment showcases the potential impacts of shortages in the region and the need for the global community to secure food aid and support for import-dependent developing countries.

### Transformative adaptation

Transformative adaptation goes beyond the gradual adjustments of incremental adaptation by aiming to reshape and fundamentally alter the systems, structures, and practices that shape society’s relationship with climate and nature. For example, reshaping and greening cities is not simply a response to climate stressors, but an ambitious rethinking of urban spaces to prioritise resilience, sustainability, and well-being for future generations, when done with equity and justice in focus^140^. By definition, transformative adaptation represents a shift in how development practices and policies are carried out, requiring new visions and pathways that change the status quo rather than reinforcing it^141^.

While reactive or incremental adaptation measures, such as improving flood defences or early warning systems, are directly supported by advances in physical climate science, upcoming practices facilitating transformative adaptation evolve around *Climate-Resilient Development* (CRD). Rather than addressing immediate climate impacts alone, CRD integrates climate adaptation with disaster risk management, climate mitigation, and sustainable development^164^. As a result, transformative adaptation switches focus from the impacts and hazards towards the underlying development context and drivers of vulnerability, which avoids exacerbating the conditions^165^. For example, the Horn of Africa drought was impactful in part because of the severity and length of the drought, but also because of shortcomings in the current drought management systems^3^. The most marginalised people, who were already grappling with chronic food and water insecurity, malnutrition, and limited access to basic services, are hit the hardest. More and better designed social protection systems may help decrease poverty and increase resilience to shocks if combined with longer-term visions on moving toward climate-resilient development, which hinges also on reducing greenhouse gas emissions and the achievement of sustainable development.

In the Caribbean, the cases of Hurricanes Irma and Maria in 2017 also demonstrated how existing vulnerability exacerbates the impact of weather events. In Puerto Rico, the hurricanes laid bare the already fragile national grid, affecting everything from homes where diabetes patients kept their insulin refrigerated to hospitals that lost power, forcing physicians to perform surgeries and emergency procedures using mobile phone flashlights^166^. The poor state in which the Puerto Rico electric power system met the storms was due to decades of mismanagement of the public energy service corporation combined with a political will to follow privatisation schemes, which proved to make the population even more vulnerable^167^. It took up to 6 months for some of the urban settlements to get re-connected, and power outages continue to disrupt both recovery and development. Meanwhile, in Sint Maarten, the remarkable speedy restoration of power to most districts of the 14 sq. mile country within less than a week was a result of the lessons learned from Hurricane Louis in 1995, after which the power company placed electric cables underground – building back better. Despite this progress, a significant portion of low-income residents (approximately 20–25%) still reside in self-organised housing that is highly susceptible to extreme weather and lacks adequate access to electricity and water^168^. Although many homes were repaired or rebuilt, those in informal settlements did not receive support^169^. A transformation in how support is being provided and to whom is required to reduce future impacts from hurricanes.

Indigenous and local knowledge hold key values in transformative adaptation for climate change^53,170^ and it is key for both researchers and decision-makers to centrally include the memory and perspectives of local and Indigenous communities across their work. As an example, the value of Indigenous knowledge and local knowledge was evident when looking at the impacts from Irma on water security. The island’s large desalination infrastructure was demolished, leaving many without water, and some have argued that traditional practices of decentralised water infrastructure may offer an alternative to incremental engineering practices, enhancing resilience to future hurricanes^171^.

Transformations include changes to underlying values, worldviews, ideologies, structures, and power relationships^164^. The transformations may be difficult but can be driven by actions and social choices from multiple actors, from policymakers to civil society, and all sectors, from education, industry, media, science and art. Social cohesion and equity, individual and collective agency, and democratising knowledge processes may drive transformational change^164^. Good governance, sustainable finance, and institutional capacity are enabling conditions that contribute to a stable pyramid.

### Identifying the right mix of measures

The three layers of options have their benefits and limitations: reactive adaptations are effective in the short term, but may not be enough to ensure future resilience. Incremental adaptation provides very concrete actions to reduce risk from climate change through, e.g., flood defences. However, these may not be as effective when not aligned with a long-term vision and the Sustainable Development Goals (SDGs) and may have negative impacts on marginalised groups (maladaptation). Transformative action is required for future resilience to extreme events and to find efficiencies across various policy domains (SDGs, Sendai Framework, Paris Agreement). However, there is no blueprint for transformative action and because it encompasses such a big topic over a long timescale, it is sometimes critiqued how to go beyond the “blah blah blah” of transformation^172^.

Just like the use of multiple lines of evidence in the “Approaches for analysing unprecedented weather” section, the three layers provide complementary actions that together encompass the right mix of measures. For example, the Pacific Northwest heatwave was captured by weather predictions, yet led to over 850 deaths despite early action and disaster response efforts^5^. This event led to questions from some organisations in the Netherlands: could this happen in our city as well?^173^ Multiple lines of evidence point to the potential of unprecedented heat near Eindhoven beyond that in the conventional observational record (Box 1). To stop being surprised by unprecedented heat in the Netherlands, therefore, requires action across the three layers.

**Fig. 4:**
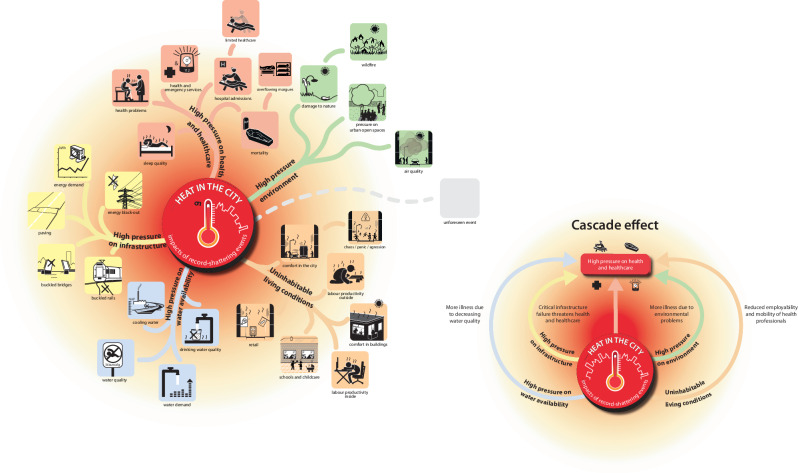
Expected impacts and cascading effects of unprecedented heat. This mindmap, based on Klok and Kluck ^176^, illustrates how unprecedented heat affects various sectors. On the left, unprecedented heat is positioned centrally, with various colours representing pressures on sectors such as the environment, living conditions, water availability, infrastructure and healthcare. On the right, the same mindmap is reorganised to illustrate that multiple pressures have cascading impacts on the city’s healthcare.

Currently, clear heat early actions are limited to yellow and orange warning levels for the national heat action plan in the Netherlands, as these were previously considered the maximum actionable levels for early response. However, discussions have started to rethink the range of possible actions beyond informing organisations that support vulnerable groups, such as introducing small-scale cooling shelters in neighbourhoods when a code red warning is issued^174^. Furthermore, disaster managers have started doing the first disaster response exercises for heat^175^. The storyline that the July 2019 heatwave could reach up to 45 °C in a 2 °C warmer world was the basis for updating a mindmap from Klok and Kluck ^176^ of keeping the city liveable in the future, towards keeping the city survivable during unprecedented heat. The mindmap shows that unprecedented heat would put high pressure on critical infrastructure throughout the city simultaneously, with cascading impacts on the city’s healthcare (Fig. 4). This mindmap is now being used in the disaster response exercises^175^.

However, the discussions around unprecedented heat mostly remain about short-term disaster preparedness and disaster response (the reactive layer). Examples of incremental adaptation in the Netherlands are the stimulation of awnings to prevent buildings from excessive heating, as well as designing urban parks as cool spaces during heatwaves^177^. Practitioners may further engage with stress testing of existing infrastructure to unprecedented heat to evaluate if improvements to existing infrastructure are necessary. Furthermore, there may be limits to the effectiveness of urban parks and awnings during unprecedented heat. Therefore, practitioners may also stimulate transformational action that moves beyond just tackling heat and may include rethinking the city to prioritise resilience, sustainability, and well-being. Solidarity, social cohesion, and equity are values that help the most vulnerable groups beyond what is possible in early actions during events. Rethinking the healthcare system is an action that relates to almost all of the SDGs, whilst climate mitigation is also crucial to be able to adapt to unprecedented heat.

## Conclusion and outlook

We are seeing ‘surprising’ weather happening around the world. The surprise lies in the weather hazard itself or in its (apparent) unexpected impact. There is a growing scientific interest and capabilities in identifying unprecedented weather through various methods, allowing us to stop being surprised by weather hazards. In this paper, we argue that four lines of evidence can provide complementary information to identify plausible examples of unprecedented weather.

In order to stop being surprised by the impacts of these events, building resilience is required at all levels. As a conceptual tool, we use the ‘adaptation pyramid’ consisting of reactive (short-term disaster preparedness and disaster response), incremental (long-term disaster prevention), and transformative adaptation strategies. Each layer plays a distinct role in strengthening resilience: while reactive measures address immediate threats, transformative adaptation builds long-term resilience. A balanced strategy, focusing on both short-term preparedness and structural transformation, is necessary to manage the intensifying impacts from unprecedented weather, with transformative adaptation as the foundation.

The methods for identifying unprecedented weather events enable us to anticipate hazards more accurately, fostering improved reactive and incremental adaptation. Future research may use principles of co-production at the core for putting the methods to identify unprecedented weather events into action. For instance, the “Exploring Unprecedented Extremes” workshop was convened in November 2023 to guide researchers not only by scientific curiosity but also by the needs of local practitioners and decision-makers^178^. The integration of these approaches with Indigenous knowledge and local knowledge will contribute to making solutions locally relevant and culturally appropriate^164^. Human and financial capacity, along with the necessary instruments and technologies, are essential to enable the uptake and implementation of the approaches.

Transformative adaptation does not tackle unprecedented weather alone, but centres around adaptive capacity and the drivers of vulnerability through aligning global goals like the SDGs, Sendai Framework, and Paris Agreement. Principles around locally-led adaptation may help the reactive and incremental adaptation actions to stimulate transformational change^179^. While climate services support reactive and incremental adaptation, future work may design climate services that support transformational adaptation^180^.

Finally, it is key that scientists and policymakers address the enabling conditions and barriers to transformative change and build resilience to unprecedented weather. Key are governance, sustainable finance, and institutional capacity to help various actors build ‘stable pyramids’. This way, we can avoid being surprised by unprecedented weather and ensure that it does not cause unprecedented impacts when it occurs.

## Supplementary information

Supplementary Information
