# Supplementary material for: How to stop being surprised by unprecedented weather
Source: Nat Commun. 2025 Mar 10;16:2382. doi: 10.1038/s41467-025-57450-0 (PMC11894206; doi:10.1038/s41467-025-57450-0)
Supplement: Supplementary file 1 — Supplementary Information [file 41467_2025_57450_MOESM1_ESM.pdf]

How to stop being surprised by unprecedented weather

Timo Kelder*[1,2], Dorothy Heinrich[3,4], Lisette Klok[1], Vikki Thompson[5], Henrique M. D. Goulart[2,6], Ed Hawkins[4], Louise Slater[7], Laura Suarez-Gutierrez[8,9], Rob Wilby[10], Erin Coughlan de Perez[11,3], Liz Stephens[3,4], Stephen Burt[4], Bart van den Hurk[6], Hylke de Vries[5], Karin van der Wiel[5], E. Lisa F. Schipper[12], Antonio Carmona Baéz[13], Ellen van Bueren[14] and Erich Fischer[8]
* contact information : *timo@climateadaptationservices.com*

1 Climate Adaptation Services Foundation (CAS), Netherlands
2 Institute for Environmental Studies, Vrije Universiteit Amsterdam, Amsterdam, Netherlands
3 Red Cross Red Crescent Climate Centre, The Hague, The Netherlands
4 Department of Meteorology, University of Reading, Reading. UK.
5 Royal Netherlands Meteorological Institute (KNMI), Netherlands
6 Deltares, Delft, the Netherlands
7 School of Geography and the Environment, University of Oxford, Oxford, UK
8 Institute for Atmospheric and Climate Science, ETH Zurich, Zurich, Switzerland
9 Institut Pierre-Simon Laplace, CNRS, Paris, France
10 Geography and Environment, Loughborough University, Loughborough, UK
11 Feinstein International Center, Friedman School of Nutrition Science and Policy, Tufts University, Boston, MA, USA
12 Department of Geography, University of Bonn, Bonn, Germany
13 University of St. Martin (USM), Phillipsburg, Sint Maarten
14 Faculty of Architecture and the Built Environment, Delft University of Technology, Delft, The Netherlands

Supplementary Materials

METHODOLOGICAL DESCRIPTION OF BOX 1 IN MAIN TEXT

Eindhoven is selected as a case study to illustrate the use of four complementary approaches to identify plausible unprecedented heat in this region. Here, we describe the methodology for these four complementary approaches:

1. Conventional methods

We assess the return period of the record daily maximum air temperature in Eindhoven at different global temperature states using observed data for the period 1951-2023 (Figure 3a). This is achieved by fitting a nonstationary GEV distribution to the historical observations, using GISTEMP smoothed global mean surface temperature as covariate (KNMI Climate Explorer, as in Philip et al., 2022[1]). We compare a

preindustrial world to the climate of 2019, 1.2 °C warmer than preindustrial. The record event in 2019 has a return period of at least 3000 years in the preindustrial world, but this has decreased to ~50 (12-10000) years in the current climate.

2. Past events

We show the reconstructed summer mean temperature timeseries for the Netherlands from 1500 to 2003 (KNMI Climate Explorer, as in Luterbacher et al. 2016[2]). According to this timeseries the most extreme, hot, summer occurred in 1540. Note that we assess summer mean temperatures here, which may not necessarily correspond with annual maximum daily temperatures studied in the other lines of evidence. In addition, we must be cautious when comparing modern temperature datasets spanning only the last few decades with longer reconstructed proxy-based data, as the variability and uncertainty in the longer datasets is likely to be larger. This will lead to an over-representation of extreme events in the distant past. For example, Orth et al., (2016)[3] suggested there is a low probability (~5%–10%) that the Central European summer of 1540 was actually hotter than 2003. It is therefore useful to incorporate multiple datasets. Other summers that stand out with agreement between multiple observational temperature records include the years of 1719, 1846, 1947, and 1976 (Supplementary Figure 1; also see Figure 2).

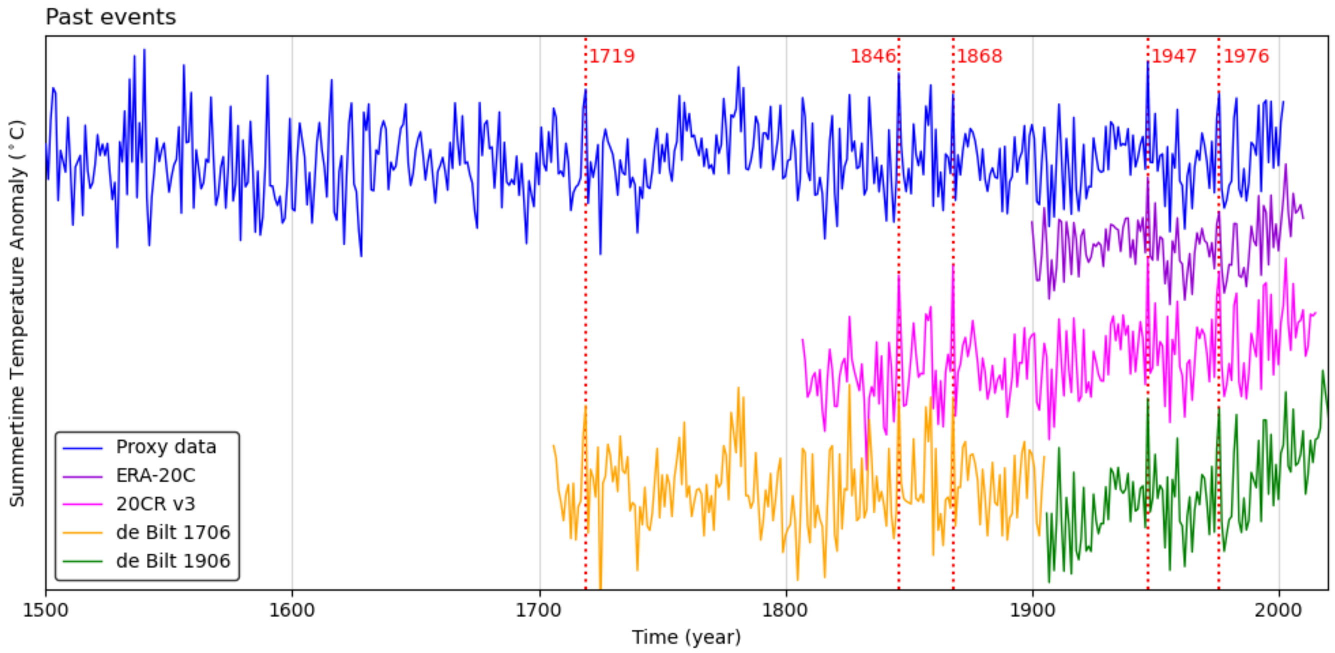

Supplementary Figure 1: Summertime (June-July-August) mean temperature anomalies for Netherlands (3.75 to 6.75 °E, 51.25 to 53.75 °N) (blue) 1500 to 2003 from KNMI Climate Explorer (Luterbacher et al., 2016[2]), (purple) 1900 to 2010 from ECMWF Reanalysis of the 20th Century ERA-20C (Poli et al., 2016[4]), (pink) 1806 to 2015 from NOAA-CIRES-DOE Twentieth Century Reanalysis V3[5] (Slivinski et al., 2019), (orange) de Bilt, 1706 to 1905 from KNMI Climate Explorer (van Engelen and Nellestijn,

1996[6]), and (green) de Bilt, 1906 to 2020 from the KNMI observational network, station number 260. Several hot years are highlighted with red dashed lines to show agreement between datasets.

3. Event-based storylines

Finally, we examine a storyline approach to the heatwave of July 2019[7]. In this approach the entire heatwave is translated into a future climate. This is done using high-resolution regional modelling in combination with the Pseudo Global Warming (PGW) approach[8]. PGW requires two regional climate model (RCM) simulations: a dynamical downscaling of the event in the current climate using reanalysis as boundary and initial conditions, and a simulation with the boundary and initial conditions perturbed by adding a future-change signal (the 'delta-field'). This delta-field has a small amplitude compared to the daily weather variability and is derived from the mean future response given a particular future scenario or global warming level. The RCM adapts to the new boundary conditions, leading to a future simulation of the event. Here we use a delta-field taken from the dry-trending KNMI'23 national climate scenarios for the Netherlands[9]. To get to "city-scale" resolution, dynamical downscaling is carried out in a two-step nested approach, using RACMO[10] at 12 km resolution and subsequently using the convection permitting regional climate model HCLIM43-AROME[11] at 2.5 km resolution. From the PGW simulation with HCLIM43-AROME (Figure 3c) we see that in a 2 °C warmer world the July 2019 heatwave would reach up to 45 °C[7]. More generally we find that maximum temperatures during heatwaves in the region typically increase 1.5 to 2.5 times faster than the imposed (global) warming level[7].

4. Climate model simulations

Next, we explore the likelihood of similar events in a 16-member ensemble with a regional climate model (RACMO, nested in EC-Earth3p5[10,12,13]). The model follows historic forcing until 2014, and SSP5-8.5 forcing from 2015. For both the model ensemble (~0.15° resolution) and ERA5 (0.25° resolution) we select the grid point in which Eindhoven is located, and compute the annual maximum of daily temperature maxima (Txx). We assess potential model bias over the period 1981-2022. The bias in the mean is corrected, model variability is close to that of ERA5 and not corrected. The resulting data shows that the likelihood of air temperature exceeding 40 °C has increased and will continue to increase under greenhouse gas forcing (Figure 3d). The highest simulated air temperature is 48 °C, near the end of the century. Note however, that the model breaches 40 °C for the first time in 2043. The simulated trend is smaller than that in ERA5, likely partially due to an underestimation of increased southerly circulation[14]. At the same time, from 2015 onwards, the model follows a high-end emission scenario which has received criticism from energy researchers[15]. The highest simulated air temperature is, therefore, likely an underestimation within the high-end emission scenario.

References

1. Philip, S. *et al.* A protocol for probabilistic extreme event attribution analyses. *Advances in Statistical Climatology, Meteorology and Oceanography* **6**, 177–203 (2020).
2. Luterbacher, J. *et al.* European summer temperatures since Roman times. *Environ. Res. Lett.* **11**, 024001 (2016).
3. Orth, R., Vogel, M. M., Luterbacher, J., Pfister, C. & Seneviratne, S. I. Did European temperatures in 1540 exceed present-day records? *Environmental Research Letters* **11**, 114021 (2016).
4. Poli, P. *et al.* ERA-20C: An Atmospheric Reanalysis of the Twentieth Century. *Journal of Climate* **29**, 4083--4097 (2016).
5. Slivinski, L. C. *et al.* Towards a more reliable historical reanalysis: Improvements for version 3 of the Twentieth Century Reanalysis system. *Quarterly Journal of the Royal Meteorological Society* **145**, 2876–2908 (2019).
6. van Engelen, A. & Nellestijn, J. *Monthly, Seasonal and Annual Means of Air Temperature in Tenths of Centigrades in De Bilt*. (1996).
7. Vries, H. de, Lenderink, G., Meijgaard, E. van, Ulft, B. van & Rooy, W. de. Western Europe's extreme July 2019 heatwave in a warmer world. *Environ. Res.: Climate* **3**, 035005 (2024).
8. Schär, C., Frei, C., Lüthi, D. & Davies, H. C. Surrogate climate-change scenarios for regional climate models. *Geophysical Research Letters* **23**, 669–672 (1996).
9. van der Wiel, K. *et al.* KNMI'23 Climate Scenarios for the Netherlands: Storyline Scenarios of Regional Climate Change. *Earth's Future* **12**, e2023EF003983 (2024).
10. Meijgaard, E. van *et al. Refinement and Application of a Regional Atmospheric Model for Climate Scenario Calculations of Western Europe*. https://library.wur.nl/WebQuery/wurpubs/427097 (2012).

11. Belušić, D. *et al.* HCLIM38: a flexible regional climate model applicable for different climate zones from coarse to convection-permitting scales. *Geoscientific Model Development* **13**, 1311–1333 (2020).
12. Döscher, R. *et al.* The EC-Earth3 Earth system model for the Coupled Model Intercomparison Project 6. *Geoscientific Model Development* **15**, 2973–3020 (2022).
13. Muntjewerf, L., Bintanja, R., Reerink, T. & van der Wiel, K. The KNMI Large Ensemble Time Slice (KNMI–LENTIS). *Geoscientific Model Development* **16**, 4581–4597 (2023).
14. Vautard, R. *et al.* Heat extremes in Western Europe increasing faster than simulated due to atmospheric circulation trends. *Nat Commun* **14**, 6803 (2023).
15. Ritchie, J. & Dowlatabadi, H. Why do climate change scenarios return to coal? *Energy* **140**, 1276–1291 (2017).
